# EHMTI-0178. CGRP monoclonal antibody LY2951742 for the prevention of migraine: a phase 2, randomized, double-blind, placebo-controlled study

**DOI:** 10.1186/1129-2377-15-S1-G10

**Published:** 2014-09-18

**Authors:** P Goadsby, D Dodick, E Spierings, J Scherer, S Sweeney, D Grayzel

**Affiliations:** 1 NIHR-Wellcome Trust Clinical Research Facility (KCL) and Dept. of Neurology (UCSF), King's College London and Univ. of California - San Francisco, London, UK; 2 Dept. of Neurology, Mayo Clinic, Phoenix, USA; 3 Dept. of Neurology (BWH) and Headache & Face Pain Program, Brigham & Women's Hospital Harvard Medical School and Tufts School of Dental Medicine, Boston, USA; 4 Eli Lilly and Company (retired), Indianapolis, USA; 5 Arteaus Therapeutics, Cambridge, USA

## Introduction

Migraine remains poorly treated with few effective preventive medications available.

## Aim

We evaluated the efficacy and safety of LY2951742, a fully humanized monoclonal antibody to Calcitonin Gene-Related Peptide (CGRP) for migraine prevention.

## Methods

Eligible subjects with 4-14 migraine headache days (MHD) per month were randomized in a double-blind manner to LY2951742 or placebo, administered subcutaneously, every other week over a 12-week period. The primary endpoint was the mean change from baseline in number of MHD in the last 28 day period (month three); secondary end points included the mean change in headache days, migraine attacks, and the 50% responder rate at month 3.

## Results

Mean change in MHD (primary) was -4.2 vs. -3.0 for LY2951742 (n=107) and placebo (n=110), respectively (p = 0.003). LY2951742 was superior to placebo for all secondary endpoints including headache days -4.9 vs. -3.7 (p = 0.0117), migraine attacks -3.1 vs. -2.3 (p = 0.0051), and 50% responder rate, 70% vs. 45% (OR 2.88 [CI 1.78;4.69]). An exploratory endpoint of complete response (100% reduction in MHD in month 3) was 31.6% vs. 17.3% (OR 2.16 [CI 1.24;3.75]) for LY2951742 and placebo, respectively. Adverse events reported by approximately ≥5% of LY2951742-treated patients and more frequently with LY2951742 than placebo included upper respiratory tract infections, injection site pain, neck pain, abdominal pain, dizziness, injection site erythema, rash, hypertension and pain in extremity.

## Conclusions

Treatment with LY2951742 demonstrated significant separation from placebo on the primary and secondary endpoints. LY2951742 appeared to be safe and well-tolerated.

